# A randomized controlled trial comparing community lifestyle interventions to improve adherence to diet and physical activity recommendations: the VitalUM study

**DOI:** 10.1007/s10654-020-00708-2

**Published:** 2020-12-30

**Authors:** Hilde Marijke van Keulen, Gerard van Breukelen, Hein de Vries, Johannes Brug, Ilse Mesters

**Affiliations:** 1 Department of Health Promotion, Maastricht University, Maastricht, The Netherlands; 2 Department of Methodology and Statistics, Maastricht University, Maastricht, The Netherlands; 3 Department of Epidemiology and Biostatistics, National Institute for Public Health and the Environment (RIVM), Utrecht RIVM and VU Medical Center, Amsterdam, The Netherlands; 4 Department of Epidemiology, Maastricht University, Maastricht, The Netherlands; 5 CAPHRI Care and Public Health Research Institute, PO Box 616, 6200 MD Maastricht, The Netherlands; 6 Department of Child Health, Now Employed by TNO, PO Box 3005, 2301 DA Leiden, The Netherlands

**Keywords:** Lifestyle, Guideline adherence, Physical activity, Fruit intake, Vegetable intake, Tailored communication, Computer-generated health communication, Motivational interviewing

## Abstract

Worldwide, adherence to national guidelines for physical activity (PA), and fruit and vegetable consumption is recommended to promote health and reduce the risk for (chronic) disease. This study reports on the effectiveness of various social-cognitive interventions to improve adherence to guidelines and the revealed adherence predictors. Participants (*n *= 1,629), aged 45–70 years, randomly selected and recruited in 2005–2006 from 23 Dutch general practices, were randomized (centralized stratified allocation) to four groups to receive a 12-month lifestyle intervention targeting guideline adherence for PA and fruit and vegetable consumption. Study groups received either four computer-tailored print communication (TPC) letters (*n *= 405), four telephone motivational interviewing (TMI) sessions (*n *= 407), a combined intervention (two TPC letters and two TMI sessions, *n *= 408), or no intervention (control group, *n *= 409). After the baseline assessment, all parties were aware of the treatment groups. Outcomes were measured with self-report postal questionnaires at baseline, 25, 47 and 73 weeks. For PA, all three interventions were associated with better guideline adherence than no intervention. Odds ratios for TPC, TMI and the combined intervention were 1.82 (95% CI 1.31; 2.54), 1.57 (95% CI 1.13; 2.18), and 2.08 (95% CI 1.50; 2.88), respectively. No pedometer effects were found. For fruit and vegetable consumption, TPC seemed superior to those in the other groups. Odd ratio for fruit and vegetable consumption were 1.78 (95% CI 1.32; 2.41) and 1.73 (95% CI 1.28; 2.33), respectively. For each behaviour, adherence was predicted by self-efficacy expectations, habit strength and stages of change, whereas sex, awareness and the number of action plans predicted guideline adherence for fruit and vegetable intake. The season predicted the guideline adherence for PA and fruit consumption. The odds ratios revealed were equivalent to modest effects sizes, although they were larger than those reported in systematic reviews. This study indicated that less resource intensive interventions might have the potential for a large public health impact when widely implemented. The strengths of this study were the participation of lower educated adults and evaluation of maintenance effects. (Trial NL1035, 2007-09-06).

## Introduction

Meeting the guidelines for fruit and vegetable consumption and physical activity (PA) lowers the risk for cardiovascular morbidity and mortality [1]. Therefore, it is recommended to refer adults with an unhealthy lifestyle to interventions that promote lifestyle change [2]. Dutch guidelines advise that individuals consume at least two servings (approximately 200 grams) of fruit and 200 grams of vegetables every day and engage in moderately intensive PA on at least five days per week, for 30 or more minutes a day [3]. Many adults, however, do not meet the public health recommendations for these behaviours. Approximately half of the Dutch general population (aged 40-75 years) is sufficiently physically active [4, 5], and about one-third meet the fruit- and vegetable recommendation [5, 6]. Therefore, interventions are needed to promote adherence to these guidelines, especially interventions that can be implemented at scale considering the population in need is significant [7].

Previous studies have indicated that theory-based computer tailoring and (telephone) motivational interviewing have the potential to reach large populations and change health behaviours [8, 9]. Hence, few studies have compared these methods in changing PA or fruit and vegetable consumption. In this study, we aim to evaluate the effects of computer-tailored print communication (TPC), telephone motivational interviewing (TMI) and a combined version of them in meeting the public health guidelines for PA and fruit and vegetable consumption. We hypothesize that TMI will outweigh TPC, since TMI provides real-time tailoring and interpersonal contact, ingredients assumed to produce a better outcome (at 18 months after baseline) [10, 11]. A detailed description of the study design can be found elsewhere [12].

Our theory-informed interventions promote health behaviour by changing behavioural determinants. Therefore, our second aim is to examine the predictors of guideline adherence in order to determine the success of the intervention.

Pedometers are often utilized to increase PA [13]. Our third aim is to examine the effects of pedometers on the adherence to the PA guideline.

Altogether, our comparative-effectiveness study contrasting three broad-reach intervention delivery modalities may help in informing the appropriate use of resources to change public lifestyle behaviour.

## Methods

### Trial design

The study participants were allocated to four groups using stratified computer randomization (Actigraph). One group received four TPC letters, one group received four TMI sessions, one group alternately received two TPC letters and two TMI sessions (combined intervention), and one group received no intervention (control group).

After the baseline assessment, treatment allocation concealment was prohibited due to the different nature of the interventions. Investigators were aware of the group assignment, but they had no in-person contact with participants during the provision of interventions. There was also no in-person contact during the self-report assessments, with the exception that some participants were phoned to collect missing data. Intervention effects were assessed by two follow-up written questionnaires (weeks 47 and 73). All letters and questionnaires were mailed to the participants’ home addresses. Two reminders were sent, if needed. Furthermore, two intermediate telephone surveys were conducted. In week 25 (after two intervention exposures), a telephone survey assessed all participants’ behaviours and behavioural determinants to gather up-to-date information for the next computer-tailored intervention and to assess the intermediate effects of the interventions. Participants in the TPC group received an additional telephone survey (week 39) to collect the most recent data on their behaviour and its determinants for the fourth tailored letter. Data entry was done by an external organization (MEMIC-Centre for data entry and management). Participants in the intervention groups received their four intervention components at 5, 13, 30 and 43 weeks after the baseline assessment.

Half of the participants in all the intervention groups were randomly selected to receive a pedometer before the third intervention component (week 29); the remainder received this device after the last follow-up. The Medical Ethics Committee of Maastricht University and the University Hospital Maastricht approved the study.

### Participants

**Fig. 1:**
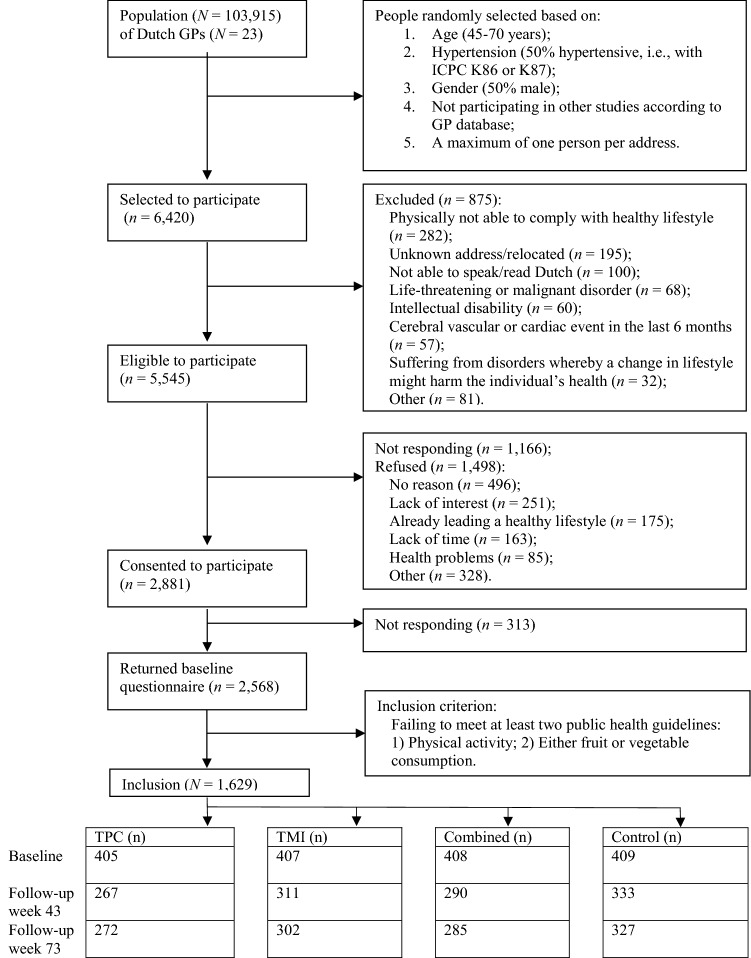
Flow diagram of the selection and enrollment of the participants. *Notes GP* general practice, *ICPC* international classification of primary care; K86 or K87 = hypertension without or with organ damage, respectively

Participants (*n *= 6420 outpatients) were randomly selected from the database of the Research Network Family Medicine Maastricht (RNFM), which contains systematically collected medical data (demographics, disease, diagnosis, and medication) of all patients from 23 Dutch general practices (GPs), reflecting Dutch primary care practice (Fig. 1) [12, 14]. Inclusion criteria were: (1) aged 45–70 years; (2) about 50% diagnosed by their GP as hypertensive according to the International Classification of Primary Care (ICPC code K86 or K87 for hypertension without or with organ damage respectively; https://www.nhg.org/themas/artikelen/icpc-online, accessed 9 September 2020); (3) about 50% male; (4) not participating in other studies according to the GP database; and (5) only one person per address. Hypertension status was included to check whether already having a risk factor for cardiovascular disease (CVD; disease awareness) moderated the effects of the intervention [15]. This is why we selected patients aged 45–70 years.

The GPs checked the suitability of the participants selected. Exclusion (*n *= 875, 14%) was due to, for example, inability to walk or inability to speak or read Dutch.

A total of 5545 people received an invitation letter explaining the study content and randomization procedures. Non-responders (*n *= 2341) received a reminder after 4 weeks. Reasons for refusing participation included”lack of interest” or “lack of time”. A total of 2881 people returned the consent form and, thereupon, received a written baseline questionnaire. Those who returned the questionnaire (*n *= 2568) received feedback on their lifestyle behaviours and were included in the randomised controlled trial (RCT) (*n *= 1629), if they failed to meet at least two of three Dutch public health guidelines (for PA and either fruit or vegetable intake). Participants were recruited and treated in batches, with 18 months elapsing between the first and last batch.

### Interventions

*TPC.* The TPCs were built on existing theory-based computer-tailored interventions, whose effectiveness have been established in earlier studies e.g., [16, 17]. They were based on the reasoned action approach [18], social cognitive theory [19], and insights from stages of change models (i.e., the transtheoretical model) [20] and implementation intentions theory [21], combined in the I-change Model [22], as well as on additional qualitative research. Study participants received stage-matched advice [23]. The tailoring variables were age, sex, awareness, attitude (pros and cons), self-efficacy expectations, action plans, stage of change and current behaviour according to the self-report questionnaire. Data on these variables were gathered with our written questionnaires. A computer algorithm connected survey items to a feedback message file in order to provide written individual feedback. The letters on PA, TPC1 and TPC2 (each 3-6 pages) were personalized with the participant’s name and included the following elements: introduction, specific behavioural feedback on targeted behaviour and related social-cognitive determinants, stage-matched advice to change behaviour and conclusions. The subsequent letters on fruit and vegetable consumption, TPC3 (2-4 pages) and TPC4 (4-6 pages) were also personalized and reinforced tailored feedback on behavioural progress and stages of change. We used a structure similar to that in TPC1 and TPC2.

*TMI.* Motivational interviewing is grounded in the similar social–cognitive theories mentioned above, which are translated into specific relational and technical counselling methods [24]. Interview protocols were derived from the Healthy Body Healthy Spirit trial and used to support treatment integrity [25]. Participants could choose the order of the conversation topics in interviews 1 and 3; if PA was preferred in interview 1, fruit and vegetable consumptions were discussed in interview 2, and vice versa. Procedures were performed as follows: giving introduction, assessing current behaviours and progress, discussing the public health guideline, assessing and enhancing motivation and self-efficacy for behaviour change, assessing readiness to change, and summarizing and closing the session. Additional topics could be discussed (e.g., the current situation and progress on action plans in subsequent interviews, the tailored letters (combined group) and values in life). Information on the training for those administering TMI and the raters of the TMI fidelity, both conducted by Master’s level students in Psychology and Health Promotion, has been described elsewhere [26]. Interviewers had MI beginner proficiency.

*Combined* The first letter and interview addressed PA, and the second letter and interview focused on fruit and vegetable consumption.

*Control* Participants received one tailored letter after the last follow-up questionnaire.

*Pedometer* The pedometer was provided with an instructional letter that encouraged participants to gradually increase their number of steps to at least 10,000 a day [14].

### Outcome measurement

The modified CHAMPS PA questionnaire was used to assess the frequency of an activity (times per week), its duration (hours per week) and intensity (e.g., walking in a leisurely vs. brisk manner) concerning a typical week during the past 4 weeks [27, 28]. The activities measured included cycling in a leisurely or brisk manner and doing light or heavy housekeeping. Metabolic equivalents (METs) were determined for each activity on the basis of the PA compendium by Ainsworth et al. [29]. MET levels were used as cut-offs to calculate the total number of weekly PA hours with at least moderate intensity. Only activities with at least three METs were considered moderate for all participants [30]. Because the modified CHAMPS cannot determine which participants are physically active with moderate intensity for at least five days a week, the summary question from the Short QUestionnaire to ASsess Health-enhancing PA (SQUASH) [31] was added: “How many days a week do you cycle, engage in do-it-yourself activities, do gardening, play a sport or engage in other strenuous physical activities for at least 30 min a day?”. Participants were only coded as meeting the PA guideline if they were physically active with at least moderate intensity for at least 2.5 h a week according to the modified CHAMPS and answered “five or more days” to the SQUASH summary question [32].

*The food frequency questionnaire (FFQ)* was used to estimate the fruit and vegetable intake [33]. Participants filled out 16 items about the frequency (days per week) and quantity (servings/serving spoons per day) of vegetables (cooked and raw) and fruit (juice, tangerines, other citrus fruits, apples or pears, bananas, and other fruits) concerning a typical week during the past 4 weeks. Frequency and quantity were used to determine daily consumption. Adherence was sufficient if participants consumed at least two servings of fruit a day and at least 200 grams of vegetables a day (four serving spoons) [34].

*Covariates in the analyses of intervention effects* were sex, hypertension status, age, highest completed level of education, marital status, work situation, native country, presence of diabetes, smoking behaviour, alcohol consumption, family history of CVD, stress, body weight and height to calculate BMI (kg/m^2^), region of residence, season at completion of baseline questionnaire, and saturated fat intake, as well as (un-)favourable behavioural beliefs, social support, descriptive normative beliefs, self-efficacy expectations, action plans, habit strength, stage of change and awareness (see Table 4 for measurement details). Awareness was based on self-rated behaviour (by asking participants whether they rate, for instance, their intake of vegetables as low or high; 1 = low to 5 = high). This score was compared to the assessment of guideline adherence. Participants were allocated to two awareness levels: overestimators (not meeting the guideline and rating vegetable intake as intermediate to high) and underestimators or realists (other).

### Sample size

At the start of this RCT, the results of similar studies were unavailable. The sample size calculation was based on an expected effect size (Cohen’s d) of 0.3, a power of 0.9, an alpha of 0.01 (multiple testing correction), an intraclass correlation of 0.02 and an average of 70 outpatients per general practice. More details were previously published [12].

### Statistical analysis

*Baseline characteristics* of the intervention groups were assessed with SPSS Inc. Released 2006. SPSS for Windows, Version 15.0. Chicago: SPSS Inc. Other analyses were done with MLWiN [35].

*Selective dropout* Selective dropout was examined (dependent variable, 0 = no; 1 = yes) with mixed logistic regression using PQL estimation. The predictors of dropout used were group, time of measurement, group by time of measurement interactions, and the baseline values of age, gender, hypertension, region, and the level of education.

*Intervention effectiveness* Separate recommended intake levels are given in the Netherlands for fruits and vegetables, as they have been found to differ in consumption circumstance and meals, as well as in their associations with health and disease [36]. Hence, separate analyses are conducted. The effectiveness of intermediate (week 25) and short-term (week 47), as well as follow-up (week 73), were analysed with mixed logistic regression using PQL estimation. These were intention-to-treat analyses, since all available measurements of all randomized participants are analysed [37] without imputation for missing measurements. The mixed model had three levels: GPs, participants, and measurements (baseline and 25, 47 and 73 weeks). GP and participant effects were included as random intercepts. Additionally, the effects of time of measurement, group and time of measurement*group were allowed to vary randomly between GPs (time, group, and time*group) or participants (time), but no significant variance was found. Thus, the reported models had random intercepts only. Socio-demographic variables, lifestyle variables, cognitive behavioural determinants, and baseline measures of the primary outcomes were included as between-subject covariates (except for the baseline behaviour of the outcome at hand, which was included as a repeated measure to allow the inclusion of patients who dropped out after the baseline measurement [38]. To the extent that these covariates are related to the outcome behaviour at hand, including them improves the power and precision of treatment-effect testing and estimation due to reduced residual outcome variance.

Having been sent a pedometer during the intervention period was included as a within-subject factor (0 = no; 1 = yes), since it was sent to participants 29 weeks after baseline, which was 1 month after the telephone survey and not yet at baseline.

In view of multiple testing, an alpha of 0.01 was used for drawing conclusions about treatment effects. Non-significant covariates (α = 0.10 to prevent type II errors) were excluded from the model, except for hypertension status (because of pre-stratification on hypertension in the randomisation), educational level, age and sex (because of hypotheses or because these variables were used to select participants) [12]. Group, time, group*time, and receiving a pedometer were never excluded, as these were the predictors of interest. Finally, the group effects on the baseline measurement of the outcome were excluded from the final model if no such differences were found (as expected, given randomized treatment assignment), because this increases power and corresponds with treating the baseline measurement as a covariate instead of as a repeated measure [38].

*Efficacy of a pedometer on PA guideline adherence* The interaction between the intervention group and pedometer was tested only when a significant pedometer effect was found, as well as significant differences between intervention groups with respect to the outcome at follow-up.

*Missing values and data checking* Participants with a missing outcome for one or more time points were included in the analyses without the imputation of missing values, using the direct likelihood approach [37]. Missing values on covariates were replaced if allowed [39] Predictors and covariates were checked for multicollinearity by inspecting their variance inflation factor (VIF). No VIFs above 10 were found, indicating the absence of multicollinearity [40].

## Results

### Baseline features

**Fig. 2:**
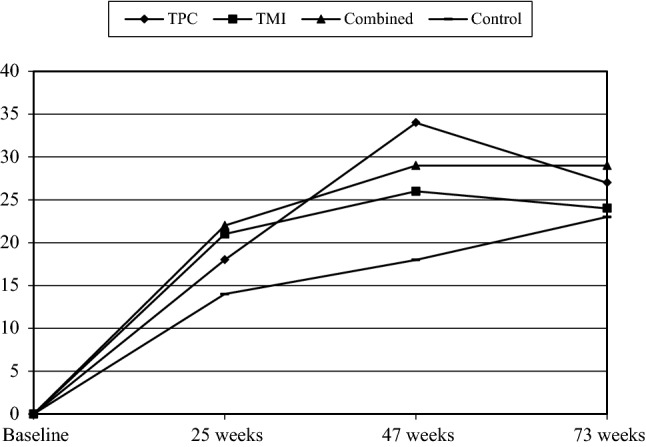
Percentage meeting the physical activity guideline per time of measurement. *TPC* tailored print communication; *TMI* telephone motivational interviewing; *Combined* combination of TPC and TMI. Figures are based on all available cases (*n* varies between time points; minor deviations were found with Figures based on complete cases). Minor deviations between plots and analyses are due to the fact that mixed logistic regression includes covariates and adjusts for selective dropout, also because results of the regression (Table 3) are presented in odds ratio’s whereas results of figures are presented in percentages. Results of the regression are decisive

**Fig. 3:**
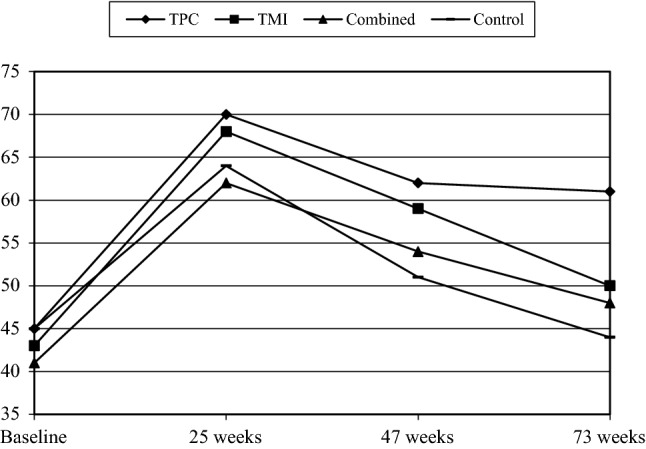
Percentage meeting the fruit consumption guideline per time of measurement. *TPC* tailored print communication; *TMI* telephone motivational interviewing; *Combined* combination of TPC and TMI. Figures are based on all available cases (*n* varies between time points; minor deviations were found with Figures based on complete cases). Minor deviations between plots and analyses are due to the fact that mixed logistic regression includes covariates and adjusts for selective dropout, also because results of the regression (Table 3) are presented in odds ratio’s whereas results of figures are presented in percentages. Results of the regression are decisive

**Fig. 4:**
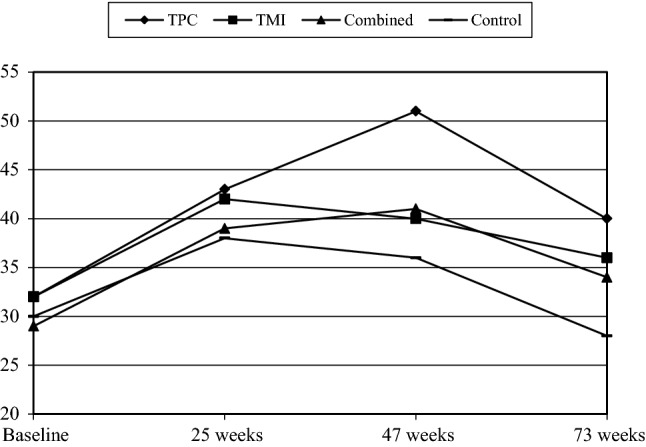
Percentage meeting the guideline for vegetable intake per time of measurement. *TPC* tailored print communication; *TMI* telephone motivational interviewing; *Combined* combination of TPC and TMI. Figures are based on all available cases (*n* varies between time points; minor deviations were found with Figures based on complete cases). Minor deviations between plots and analyses are due to the fact that mixed logistic regression includes covariates and adjusts for selective dropout, also because results of the regression (Table 3) are presented in odds ratio’s whereas results of figures are presented in percentages. Results of the regression are decisive

Table 1 entails the baseline characteristics of the participants. Table 2 and Figs. 2, 3, 4 (available online) show the percentages of participants that adhered to a guideline per group and time of measurement. There were no significant differences between the groups at baseline on outcome variables or potential covariates (all *p *> 0.05). None of the participants met the PA guideline at baseline due to the inclusion criterion, whereas 44% and 31% of the participants adhered to the guideline for fruit and vegetable intake, respectively. The average age was 57.15 years (SD = 7.13), 55% were men, and 52% were classified as hypertensive; 54% had a low educational level, while 23% had an intermediate educational level.

**Table 1:** Participants’ means (SD) or percentages regarding the Vitalum baseline variables

Variables	TPC (*n *= 405)	TMI (*n *= 407)	Combined (*n *= 408)	Control (*n *= 409)
Sex (% male)	55.8	56.5	52.5	57.2
Age (45-70 years)	57.6 (7.2)	57.3 (7.1)	56.9 (7.1)	56.8 (7.1)
Native country (% the Netherlands)	95.3	95.3	94.1	94.6
Region (% southern Limburg)	61.7	60.9	65.7	61.6
Season at completion of baseline questionnaire				
% spring; summer	74.8; 9.4	74.9; 9.3^†^	74.8; 9.8	75.1; 9.5
% autumn; winter	2.5; 13.3	2.7; 13.0^†^	2.2; 13.2	2.2; 13.2
Education level (% low; intermediate; high)	54.6; 22.1; 23.3	53.7; 23.8; 22.5	55.9; 23.2; 20.9	51.7; 24.4; 23.9
Marital status (% married or living together)	78.3	82.0	80.5	78.4
Work situation (% paid job)	46.5	47.0	48.2	52.2
Hypertension (% hypertensive)	52.1	52.1	51.7	51.3
Diabetes (% diabetic)	9.2	10.4	11.1	9.4
Perceived stress				
% less than normal; normal	14.4; 53.2	15.5; 50.2	17.5; 46.4	12.3; 51.0
% a little more; a lot	22.1; 10.2	19.7; 14.5	23.0; 13.1	25.5; 11.3
CVD family history (% no family history)	45.2	41.5	37.5	42.0
Body mass index (kg/m^2^; 15.2–46.7)	27.6 (4.3)	27.6 (4.5)	27.5 (5.0)	27.1 (4.4)
Smoking behavior (% nonsmokers)	76.2	79.8	78.6	78.6
Alcohol consumption (% non-drinkers; drinkers meets guideline; does not meet guideline)	34.7; 51.9; 13.5	39.0; 45.9; 15.1	39.9; 46.3; 13.9	38.7; 48.3; 13.0
Saturated fat intake score (2.0–37.0)	18.0 (6.0)	17.6 (5.7)	17.7 (6.1)	17.8 (5.9)
PA				
Awareness (% overestimating PA)	61.3	58.1	58.3	60.8
Attitudes				
Pros (13–65)	49.1 (7.8)	49.3 (7.3)	49.0 (7.0)	49.0 (7.0)
Cons (11–55)	38.3 (6.8)	38.9 (6.3)	38.1 (6.7)	39.0 (6.5)
Social influence				
Support (5–25)	14.7 (4.1)	14.5 (4.1)	14.6 (3.9)	14.0 (3.8)
Modelling (3–15)	9.6 (2.6)	9.6 (2.8)	9.3 (2.5)	9.5 (2.5)
Self-efficacy expectations (11–55)	36.7 (7.9)	37.2 (7.5)	36.3 (7.9)	36.8 (7.2)
Number of action plans (0–6)	2.3 (1.1)	2.2 (1.0)	2.2 (1.0)	2.3 (1.0)
Habit (3–15)	10.7 (2.7)	10.5 (2.8)	10.4 (2.7)	10.6 (2.6)
Stages (1–6)	4.1 (2.0)	4.1 (1.9)	4.0 (2.0)	4.1 (1.9)
Vegetable intake				
Awareness (% overestimating intake)	86.4	84.9	85.7	85.3
Attitudes				
Pros (8–40)	29.8 (4.1)	29.8 (4.3)	29.7 (4.6)	29.6 (4.3)
Cons (8–40)	30.7 (4.9)	30.8 (4.7)	30.7 (4.8)	31.0 (4.4)
Social influence				
Support (5–25)	14.1 (4.0)	13.9 (4.1)	14.3 (4.1)	13.6 (4.0)
Modelling (3–15)	9.9 (1.8)	9.9 (2.0)	10.0 (1.9)	9.9 (1.9)
Self-efficacy expectations (9–45)	34.5 (5.5)	34.1 (5.5)	33.8 (6.0)	34.3 (5.5)
Number of action plans (0–6)	2.1 (1.0)	2.2 (1.0)	2.1 (1.0)	2.2 (1.1)
Habit (3–15)	12.1 (2.4)	12.1 (2.4)	12.1 (2.4)	12.1 (2.2)
Stages (1–6)	4.7 (1.8)	4.7 (1.8)	4.6 (1.9)	4.7 (1.8)
Fruit intake				
Awareness (% overestimating intake)	56.8	58.8	54.4	57.5
Attitudes				
Pros (8–40)	28.7 (4.7)	28.8 (4.6)	28.5 (4.6)	28.1 (4.7)
Cons (4–20)	15.2 (2.5)	15.0 (2.6)	14.9 (2.7)	15.0 (2.6)
Social influence				
Support (5–25)	13.6 (3.9)	13.2 (3.9)	13.6 (4.0)	13.0 (4.0)
Modelling (3–15)	9.5 (1.9)	9.3 (2.0)	9.5 (2.0)	9.4 (2.0)
Self-efficacy expectations (10–50)	38.2 (6.2)	37.4 (6.3)	37.3 (7.2)	37.6 (6.7)
Number of action plans (0–5)	1.9 (0.9)	1.8 (0.9)	1.8 (0.9)	1.9 (0.9)
Habit (3–15)	10.2 (3.2)	10.1 (3.3)	10.1 (3.4)	10.0 (3.2)
Stages (1–6)	4.2 (1.9)	4.0 (1.9)	4.0 (2.0)	4.2 (1.9)

**Table 2:** Percentages (*n* of *n* total) of participants meeting a guideline per group and time of measurement, and p values of baseline group comparisons

Outcomes		Baseline	Week 25	Week 47	Week 73	p*
PA	TPC	0 (0 of 405)	18 (66 of 376)	34 (91 of 266)	27 (73 of 272)	–
	TMI	0 (0 of 407)	21 (76 of 369)	26 (82 of 310)	24 (71 of 302)	–
	Combined	0 (0 of 408)	22 (81 of 370)	29 (84 of 290)	29 (83 of 285)	–
	Control	0 (0 of 409)	14 (54 of 393)	18 (61 of 332)	23 (74 of 327)	–
Fruit intake	TPC	45 (172 of 380)	70 (263 of 376)	62 (165 of 267)	61 (165 of 272)	0.53
	TMI	43 (165 of 386)	68 (250 of 369)	59 (181 of 307)	50 (150 of 302)	–
	Combined	41 (157 of 385)	62 (227 of 369)	54 (152 of 284)	48 (137 of 285)	–
	Control	45 (177 of 391)	64 (249 of 392)	51 (166 of 326)	44 (144 of 327)	–
Vegetable intake	TPC	32 (128 of 400)	43 (160 of 376)	51 (136 of 267)	40 (109 of 272)	0.65
	TMI	32 (131 of 406)	42 (155 of 369)	40 (123 of 310)	36 (108 of 302)	–
	Combined	29 (116 of 404)	39 (143 of 370)	41 (119 of 290)	34 (97 of 285)	–
	Control	30 (122 of 404)	38 (148 of 392)	36 (119 of 332)	28 (93 of 327)	–

Figures are based on all available cases (n varies between time points)

*PA* physical activity, *TPC* tailored print communication, *TMI* telephone motivational interviewing, *Combined* combination of TPC and TMI

*Chi square tests (adherence) for comparisons between treatment groups at baseline

### Selective dropout

Of the 1629 participants, 1509 (93%) finished the intermediate survey, 1201 (74%) completed follow-up 1 and 1186 (73%) completed follow-up 2. In the TPC group, the additional survey (week 39) was completed by 356 participants (88%) (Fig. 1).

Dropout was found to be unrelated to age, sex, hypertension, or region. There were more dropouts among participants with a low educational level (i.e., less than secondary or vocational education) than among participants with a higher educational level (25% vs. 17%). It should be noted that possible bias due to group and education effects on dropout was adjusted for in the effect analyses by including all dropouts and all predictors of dropout in the analyses of each outcome.

### Efficacy of TPC, TMI and the combined version

Table 3 shows the mixed logistic regression analysis in which the outcome difference between every two groups was estimated at each time point and translated into an odds ratio with a confidence interval. The effects in Table 3 suggest that differences between groups were fairly constant over time points, except for a larger effect of TPC in week 47 (PA and vegetables) and week 73 (fruit). This table, therefore, also reports the pairwise differences based on a model that assumed constancy of differences over time points.

**Table 3:** Odds ratios (95% confidence intervals) of group comparisons in meeting the guideline per measurement, and overall values for all follow-up measurements

Out-come	Comparison^1^	Week 25	Week 47	Week73	Overall^2^
PA^a^	TPC-Control	1.53 (0.93; 2.52)	2.98 (1.80; 4.92)**	1.37 (0.83; 2.25)	1.82 (1.31; 2.54)**
	TMI-Control	1.87 (1.15; 3.04)^†^	1.74 (1.05; 2.88)^†^	1.19 (0.73; 1.96)	1.57 (1.13; 2.18)*
	Combined-Control	2.11 (1.31; 3.43)*	2.46 (1.50; 4.05)**	1.77 (1.09; 2.87)^†^	2.08 (1.50; 2.88)**
	TPC-Combined	0.72 (0.46; 1.15)	1.21 (0.77; 1.91)	0.77 (0.48; 1.24)	0.87 (0.64; 1.19)
	TMI-Combined	0.88 (0.49; 1.59)	0.71 (0.45; 1.12)	0.68 (0.42; 1.08)	0.75 (0.56; 1.02)
	TPC-TMI	0.82 (0.52; 1.31)	1.71 (1.08; 2.70)^†^	1.14 (0.71; 1.85)	1.16 (0.85; 1.58)
Fruit^b^	TPC-Control	1.52 (1.03; 2.24)^†^	1.83 (1.10; 3.02)^†^	2.45 (1.49; 4.03)**	1.78 (1.32; 2.41)**
	TMI-Control	1.31 (0.90; 1.92)	1.76 (1.09; 2.86)^†^	1.53 (0.95; 2.48)	1.44 (1.08; 1.93)^†^
	Combined-Control	na	1.31 (0.81; 2.13)	1.33 (0.82; 2.16)	1.17 (0.84; 1.62)
	TPC-Combined	1.52 (1.03; 2.24)^†^	1.39 (0.85; 2.29)	1.85 (1.13; 3.03)^†^	1.57 (1.17; 2.12)*
	TMI-Compobined	1.31 (0.90; 1.92)	1.34 (0.84; 2.16)	1.15 (0.72; 1.86)	1.27 (0.95; 1.70)
	TPC-TMI	1.31 (0.88; 1.94)	1.06 (0.65; 1.73)	1.64 (1.01; 2.65)^†^	1.34 (1.00; 1.80)
Vegetables^c^	TPC-Control	1.28 (0.88; 1.87)	2.90 (1.74; 4.83)*	2.06 (1.22; 3.46)*	1.73 (1.28; 2.33)**
	TMI-Control	1.25 (.086; .1.82)	1.51 (0.92; 2.48)	1.62 (0.97; 2.71)	1.32 (0.98; 1.79)
	Combined-Control	na	1.73 (1.05; 2.85)^†^	1.50 (0.90; 2.51)	1.31 (0.93; 1.84)
	TPC-Combined	1.28 (0.88; 1.87)	1.68 (1.01; 2.77)^†^	1.37 (0.83; 2.28)	1.42 (1.05; 1.91)^†^
	TMI-Combined	1.25 (0.86; 1.82)	0.88 (0.54; 1.42)	1.08 (0.65; 1.78)	1.08 (0.80; 1.45)
	TPC-TMI	1.10 (0.75; 1.60)	1.94 (1.19; 3.17)*	1.29 (0.78; 2.12)	1.34 (1.00; 1.80)

*PA* physical activity, *TPC* tailored print communication, *TMI* telephone motivational interviewing, *Combined* combination of TPC and TMI

†*p* < 0.05; *p < 0.01; **p < 0.001

^1^The second group is the reference category

^2^The overall odds ratio is the average of weeks 25, 47 and 73. For PA, this simplified model was obtained by dropping all group by time terms, retaining only the group and time main effects (remember that for PA, the baseline measurement was left out of the model). For fruit and vegetables, the model was obtained by replacing in all group by time interaction terms the three time dummies with a single indicator for post-test (0 at baseline, 1 at all other time points). To allow mean outcome change over time, the time dummies were kept as main effects

^a^The baseline measurement was not included in the analysis, because there were no group differences at baseline (all participants failed to meet the PA guideline at baseline). The models were adjusted for main effects week 47, week 73, whether participants received a pedometer, season, age, sex, hypertension, level of education, awareness, self-efficacy expectations, habit and stages of change. The random intercept for GP was not significant (*p* was not estimated because the effect was too small), whereas the random intercept for participant was significant (*p *< 0.001)

^b^Adjusted for main effects week 25, week 47, week 73, whether they received a pedometer, season, age, sex, hypertension, level of education, presence of diabetes, perceived stress level, awareness, self-efficacy expectations, habit, number of action plans, stages of change, PA (multiple items) and vegetable consumption (multiple items). The random intercept for GP was not significant (*p *= 0.95), however, the random intercept for participant was significant (*p *< 0.001)

^c^Adjusted for main effects week 25, week 47, week 73, dummies at baseline for TPC, TMI and their combination, whether participants received a pedometer, age, sex, hypertension, level of education, marital status, native country, family history of cardiovascular disease, awareness, modelling, self-efficacy expectations, habit, number of action plans, stages of change, fruit consumption (multiple items) and smoking behaviour. The random intercept for GP was not significant (*p *= 0.09), but the random intercept for participant was significant (*p *< 0.001)

Concerning PA guideline adherence, pairwise comparisons revealed that, after baseline, more participants in the TPC, TMI and combined group adhered to the PA guideline than participants in the control group. Although pairwise comparisons in Table 3 indicated that differences between intervention groups were not significant, the following ranking (based on the size of the odds ratio) seemed to apply: combined ≥ TPC ≥ TMI > control (with ‘>’ representing a significant difference and ‘≥’ representing a borderline or no significant difference).

For fruit consumption, pairwise comparisons showed that participants in the TPC group were more likely to adhere to the fruit consumption guideline than participants in the control group, and more participants in the TPC group met this guideline than participants in the combined group (Table 3). Participants in the TMI group appeared more likely to meet this guideline than participants in the control group (borderline significance, Table 3). The following ranking seemed to apply: TPC ≥ TMI ≥ combined ≥ control.

Regarding vegetable consumption, pairwise comparisons indicated that more participants in the TPC group adhered to the vegetable consumption guideline than participants in the combined or control group (Table 3), with the following ranking: TPC ≥ TMI = combined ≥ control.

Examining whether the treatment effects depended on educational level and hypertension status in view of the expected superiority of TMI over TPC for participants with a low educational level and without hypertension [12], no significant treatment by time by education or treatment by time by hypertension interaction was found.

### Predictors of guideline adherence

Baseline variables that significantly predicted guideline adherence in week 73 (follow-up 2) are reported in Table 4. Concerning PA, self-efficacy expectations, habit strength and stages of change positively predicted adherence, and participants who filled out the baseline questionnaire in the winter were more likely to adhere than participants who did so in the spring.

**Table 4:** Odds ratios (OR) and 95% confidence intervals (95% CI) of significant baseline covariates that predicted adherence

Outcome	Covariate	Operationalization	OR (95% CI)
PA	Season–summer	Season at completion of baseline questionnaire; 3 dummies for summer, autumn and winter (spring is reference category)	0.94 (0.65 to 1.37)
	Season–autumn		0.68 (0.30 to 1.53)
	Season–winter		1.64 (1.20 to 2.23)*
	Self-efficacy expectations	Sum of 11 items (α = .91) in which participants were asked to what extent they think they are able to meet the PA guideline in general and in high-risk situations (1 = certainly not able 5 = certainly able)	1.03 (1.01 to 1.05)*
	Habit strength	Sum of 3 items (α = .86), e.g. “Being physically active on at least 5 days a week for 30 or more minutes a day is something I do frequently” (1 = completely disagree to 5 = completely agree)	1.08 (1.02 to 1.14)*
	Stages of change	1 = “I have no plans to execute the behaviour” (not motivated) to 6 = “I have been executing the behaviour for longer than 6 months” (maintainer)	1.18 (1.09 to 1.27)**
Fruit	Season–summer	Season at completion of baseline questionnaire; 3 dummies for summer, autumn, and winter (spring is reference category)	0.94 (0.66 to 1.34)
	Season–autumn		0.64 (0.32 to 1.31)
	Season–winter		1.56 (1.13 to 2.15)*
	Age	Years	1.02 (1.00 to 1.04)^†^
	Sex	0 = man; 1 = woman	1.51 (1.20 to 1.89)**
	Awareness	0 = underestimator or realist; 1 = overestimator; awareness was based on self-rated behaviour (by asking participants whether they rated their intake of fruit as low or high; 1 = low to 5 = high) and was compared to the guideline adherence assessed by the self-report measures. Participants were allocated to two awareness levels: overestimators (not meeting the guideline and rating fruit intake as intermediate to high) and underestimators or realists (other).	0.64 (0.50 to 0.83)**
	Self-efficacy expectations	Sum of 10 items (α = .93) in which participants were asked to what extent they think they are able to meet the fruit guideline in general and in high-risk situations (1 = certainly not able 5 = certainly able)	1.04 (1.01 to 1.07)*
	Habit strength	Sum of 3 items (α = .94), e.g. “Eating fruit is something I do frequently” (1 = completely disagree to 5 = completely agree)	1.29 (1.22 to 1.36)**
	Number of action plans	Number of ticked plans (0-5)	1.19 (1.04 to 1.35)*
	Stages of change	1 = “I have no plans to execute the behaviour” (not motivated) to 6 = “I have been executing the behaviour for longer than 6 months” (maintainer)	1.25 (1.16 to 1.35)**
	PA	Hours per week	1.24 (1.10 to 1.39)**
	Vegetable consumption	Grams a day	1.00 (1.00 to 1.00)^†^
Vege-tables	Sex	0 = man; 1 = woman	1.41 (1.11 to 1.81)*
	Education level–intermediate	1 = low; less than secondary or vocational education; 2 = intermediate; secondary through pre-university education; and 3 = high; professional or university education	1.51 (1.13 to 2.02)*
	Education level–high		2.15 (1.60 to 2.89)**
	Marital status	0 = single, divorced, widowed; 1 = married or living together	1.40 (1.03 to 1.91)^†^
	Native country	0 = other than the Netherlands; 1 = the Netherlands	0.58 (0.35 to 0.96)^†^
	Family history of cardiovascular disease	0 = no; 1 = yes	1.36 (1.08 to 1.71)^†^
	Awareness	0 = underestimator or realist; 1 = overestimator; awareness was based on self-rated behaviour (by asking participants whether they rated their intake of vegetables as low or high; 1 = low to 5 = high) and was compared to the guideline adherence assessed by the self-report measures. Participants were allocated to two awareness levels: overestimators (not meeting the guideline and rating vegetable intake as intermediate to high) and underestimators or realists (other).	0.34 (0.24 to 0.49)**
	Modelling	Sum of 3 items (α = .68) in which participants were asked whether important others (partner, family or friends) executed the behaviour according to the guideline (1 = completely disagree tot 5 = completely agree; the value of 6 for ‘not applicable’ was replaced by a 3)	0.91 (0.85 to 0.98)^†^
	Self-efficacy expectations	Sum score of 9 items (α = .90) in which participants were asked to what extent they think they are able to meet the vegetable guideline in general and in high-risk situations (1 = certainly not able 5 = certainly able)	1.09 (1.06 to 1.13)**
	Habit strength	Sum of 3 items (α = .91), e.g. “Eating vegetables is something I do frequently” (1 = completely disagree to 5 = completely agree)	1.20 (1.12 to 1.29)**
	Number of action plans	Number of ticked plans (0-6)	1.16 (1.04 to 1.30)*
	Stages of change	1 = “I have no plans to execute the behaviour” (not motivated) to 6 = “I have been executing the behaviour for longer than 6 months” (maintainer)	1.34 (1.23 to 1.46)**
	Fruit consumption	Servings a day	1.46 (1.18 to 1.81)**
	Smoking behaviour	0 = not smoking; 1 = smoking occasionally or regularly	0.64 (0.48 to 0.86)*

^1^Values were based on the model which assumed constancy of differences over time points (final column in Table 3)

95% CI = 95% confidence interval

*PA* physical activity, *OR* odds ratio

†*p* < 0.05; *p < 0.01; **p < 0.001

For fruit consumption, age, self-efficacy expectations, habit strength, number of action plans, stages of change, PA and intake of vegetables were positive predictors of adherence. In addition, participants who filled out the baseline questionnaire in the winter, women and underestimators or realists were more likely to adhere to the fruit guideline than those who filled out the measurement in the spring, men and overestimators.

With regard to vegetable intake, self-efficacy expectations, habit strength, number of action plans, stages of change and fruit intake positively predicted adherence, whereas modelling negatively predicted adherence. Women; intermediately and highly educated participants; those who were married or living together; participants who were born outside the Netherlands or who had family history of CVD; underestimators; realists; and non-smokers were more likely to adhere than men; poorly educated participants; participants who were single; divorced or widowed; those who were born in the Netherlands or who had no family history of CVD; overestimators; and smokers.

### Pedometer effects

There were no differences in adherence to the PA guideline (*OR* = 0.98, 95% *CI* = 0.75; 1.28) between participants in the intervention groups who were or were not sent a pedometer.

## Discussion

This paper described the comparative effects of TPC, TMI and a combined version on adherence to the Dutch public health guidelines for PA, and fruit and vegetable consumption, which were measured with self-report questionnaires. Although TMI was expected to be most successful, TPC, TMI and the combined version were found to be equally effective in increasing the proportion of participants reporting PA guideline adherence. TPC seemed most suited in promoting adherence to both fruit and vegetable consumption guidelines. Previous analyses of our interventions concerning absolute changes in these behaviours indicated that all interventions affected PA and the dietary behaviours equally well [41]. Overall improvements were modest but comparable or better to other studies on multiple risk behaviour interventions addressing guideline adherence [42], though most assessments so far were done at 12 months at the latest [43].

This is not the first study on lifestyle change describing the relative better effectiveness of print-mediated compared to telephone-mediated programmes [44]. In any case, regarding combined fruit and vegetable consumption, a systematic review also revealed smaller effects of MI interventions compared to other programmes [45].

What could explain the superior effect of TPC in changing nutritional behaviour? TPC mailed to the participants’ home addresses can be kept and re-read, which may be important for behavioural change [46]. Of the study participants who received the letters, about 75% reported to have kept them and 50% to have read them more than once [41]. For TMI participants, recalling what was discussed and decided might be more difficult after the telephone interventions and might have impeded behaviour change. Previous studies have also found that patients may not correctly recall much of the recommendations and information given by their counsellors [47]. Furthermore, with TPC, the detail of the information is pre-set, whereas in TMI, the detail depends on the client’s conversation, causing more variability in the information provided. Maybe, the information provided in TMI was less comprehensive. Also, addressing two nutritional behaviours in one 20-minute session could have hindered a profound discussion, which may have impaired the effectiveness of TMI.

Finally, the qualification of the motivational interviewers may also provide an explanation why TMI lagged behind compared to TPC, because in our study interviewers had beginner proficiency—higher competency and more experience is expected to result in better TMI outcomes [26]. By way of conclusion, we found that, TMI helped participants to reach the intended cut-off for PA adequacy. Although, in the current form, it may be less suitable to address fruit or vegetable consumption.

As in our study, meta-analyses have reported that the effects of computer tailoring and motivational interviewing mostly manifest themselves in the short or medium term [11, 48]. Adherence rates in the study intervention groups seemed to have stabilized for PA and declined for fruit and vegetables intake at the 73-week follow-up compared to those at the 47-week follow-up. This finding also applied to our absolute behavioural improvement [41]. The type of behaviour may offer an explanation—PA may provide people with more direct physical or psychological reinforcement, which better stimulates PA maintenance (e.g., feelings of vigour or relaxation) compared to that for the intake of fruit and vegetables [49] [50].

We revealed a large increase in the number of participants who met a certain guideline from the baseline to the intermediate measurement (week 25). This could be due to the fact that this measurement was executed by telephone, which may be more subject to social desirability bias than a written questionnaire [51]. A similar increase from baseline to the intermediate telephone survey was also found in the control group. Besides the social desirability aspect, this may also indicate that merely participating in a study that requires completion of self-reported assessments can already induce behaviour change, a finding that has been reported before [52].

The second goal of this study was to investigate predictors of PA and fruit and vegetable consumption. In line with an umbrella systematic literature review, we revealed that none of our baseline sociodemographic variables predicted our PA outcome [53], although season (winter) might be a variable to account for. Also, others have mentioned the relevance of season [54]. Habit, self-efficacy and stage of change have been found as consistent variables related to PA [55, 56].

For both fruit and vegetable consumption, women were more likely to reach this lifestyle advice. For fruit consumption, age was also positively related to reaching the recommendation. A seasonal influence was found for fruit intake; however, others have reported no such influence [57]. For vegetable consumption, higher educational level, being native Dutch, having a partner and a history of CVD predicted higher intake. We observed no seasonal influence for vegetable intake, although others did reveal such a link [57]. Both fruit and vegetable norm behaviour had identical social cognitive predictors (awareness, self-efficacy, habit strength, number of action plans and stages of change), except for modelling. The limiting role of social modelling was restricted to vegetable intake only. Self-efficacy beliefs and habits have been considered in a review as variables that are consistently related with fruit and vegetable intake [58]. Other variables were also revealed in individual studies, such as the predictive value of action plans concerning fruit intake [59] or stages of change regarding fruit and vegetable consumption [60].

Finally, we examined the efficacy of a pedometer on adherence to the PA guideline. Although using a pedometer may be associated with increased PA [13], in this study, this device did not affect adherence to the PA guideline. Also, we have not found it to affect absolute change in PA [41]. Current evidence has not provided conclusive proof for its effectiveness as well [61]. Contamination may have led to a type II error. In the Netherlands, people may already possess a pedometer as a result of marketing or free gifts with food products. Moreover, this lack of effect could be explained by the fact that participants were not asked to report steps-data, and therefore, people were less motivated to use it [13]. Put differently, the positive effect of pedometers in research studies may be artificial when participants know that their steps will be evaluated.

### Limitations

Counsellors of the TMI sessions were not blinded. However, the risk of bias was probably low, since our counsellors for the TMI sessions were trained, followed an interview protocol, sessions were recorded and rated by objective assessors and the counsellors had neither inherent allegiance nor conflict of interest with the treatment provided in the study. Also, the blinding of researchers and participants was not feasible in our lifestyle study. Researchers were aware of group assignments, because they were responsible for the logistics of the project. This entailed the organization of the processing, printing and mailing of the tailored letters; the training and monitoring of both the TMI counsellors and TMI coders; the scheduling of telephone sessions; and the organization of the self-reported assessments. During the ongoing study, in-person contact between the researchers and study participants was rare; therefore, we estimated the influence of researchers on the performance of participants as negligible. Our participants were unaware of the trial’s hypothesis, and we were able to conceal their group allocation until after the baseline assessment. Furthermore, our self-reported written assessments, which were completed independently by participants at home, made interference by the researchers unlikely. Besides, data entry was done by an external organization. Nevertheless, empirical studies have shown that if true blinding is lacking, subjective outcomes effect estimates may be exaggerated [65].

Dropout was higher among participants who received a tailored letter (TPC and combined group) compared to that among those who did not (TMI and control group), as well as higher among participants with a low versus intermediate or high educational levels. Although the mixed logistic regression analyses could be biased in the case of non-ignorable dropout (i.e., dropout depending on unmeasured outcome variables, known as MNAR missingness), the analyses were intention-to-treat [37], including all available data from dropout. Treatment group and educational level were always included as predictors in the outcome analyses, and dropout did not depend on other covariates or measured outcome variables. Thus, at least under the assumption of missingness at random (MAR), the present analyses were unbiased.

As in most lifestyle interventions studies [62], we used self-report measures to assess PA, and fruit and vegetable intake. These measures are similar to the ones used to estimate lifestyle prevalence in communities for national databases. Naturally, such measures have limitation (e.g., they require the participants to have good memories and estimation skills) [63]. In addition, measuring PA in relatively older adults requires extra attention to the time frame used (i.e., they experience more memory difficulties), frequency (i.e., they are active on a more irregular basis) and type of activities that are performed by our study group (i.e., moderate intensity activities are more common in this age group) [27]. Furthermore, overestimation of the measured behaviours is likely [5]. But, for evaluation purposes, the responsiveness to change of an instrument is most relevant. It is known that our food frequency instrument has adequate responsiveness [34]. The modified CHAMPS is a valid and reliable instrument specifically for older adults and has been shown to be sensitive to change as well [27]. We had to add one (SQUASH) item to calculate adherence to the PA guideline. This item came from a validated questionnaire [31]. But because it concerned a summary question, it may have lowered the psychometric quality and may have been more prone to measurement error. This is because such questions may estimate behaviour less precisely than multiple-item questionnaires [63].

Measuring multiple behaviours and their determinants with self-report questionnaires requires considerable time investments from participants, which may have led to annoyance and thus dropouts or invalid results [64]. However, the measurement responses were adequate (93%, 74% and 73% in the intermediate and first and second follow-up measurements, respectively), but some participants only partially completed questionnaires, necessitating a call to them to complete the data collection.

Data analysis was conducted by one researcher (HvK) who was aware of group assignment. Capacity limitations did not allow us to appoint two independent data analysts (one being blind to group allocation). To avoid bias caused by the flawed analysis and interpretation of the data, the trial was analysed in accordance with a pre-specified (unchanged) protocol [12], and detailed documentation was kept for each step of the analysis. These steps were checked and discussed regularly with two members of the research team (IM and GvB). Furthermore, the scientific committee of the grant organization and the co-authors were involved in challenging the outcomes for alternative interpretations.

Our trial was funded by a national funding organization (ZonMw), the design was published before the publication of the results, the trial protocol was registered online, and the study was monitored by a medical ethics committee. All these sources of information allowed for confirmation that all primary outcomes were reported in our study publication.

### Recommendations

Following the recommendations on PA and fruit and vegetable intake have been shown to reduce the risk for CVD complications. Because the present study indicates that effects on guideline adherence may differ from absolute change, we recommend that future studies examine intervention effects both on absolute improvement and guideline adherence to choose an intervention with the most impact. Research comparing the effects of TPC and TMI is needed in a longer term measurement (> 12–8 months post-intervention) [65] to assess whether research designed to increase and promote behaviour change maintenance is needed [62]. Based on the findings of this paper, TPC is preferred over TMI or a combined version as the method to promote guideline adherence for fruit and vegetable consumption, whereas all three interventions are recommended to stimulate adherence to the PA guideline. Still, more research is necessary to confirm the advantage of TPC over the treatment modalities for adherence to the guidelines for fruit and vegetable consumption. In addition, participants with lower self-efficacy expectations, who are less motivated to change and have lower habit strength, will need more attention in future interventions to increase their adherence to guidelines for PA and fruit and vegetable consumption. This also applies to overestimators and men with regard to adherence to the fruit and vegetable consumption guidelines. Furthermore, future interventions targeting adherence to these latter guidelines should stimulate participants to formulate action plans.
